# A Convenient Synthesis of Pentaporphyrins and Supramolecular Complexes with a Fulleropyrrolidine

**DOI:** 10.3390/molecules24173177

**Published:** 2019-09-01

**Authors:** Joana I. T. Costa, Andreia S. F. Farinha, Filipe A. Almeida Paz, Augusto C. Tomé

**Affiliations:** 1 QOPNA and LAQV-REQUIMTE, Department of Chemistry, University of Aveiro, 3810-193 Aveiro, Portugal; 2 King Abdullah University of Science and Technology (KAUST), Water Desalination and Reuse Center (WDRC), Division of Biological and Environmental Sciences (BESE), Thuwal, Saudi Arabia; 3 Department of Chemistry, CICECO—Aveiro Institute of Materials, University of Aveiro, 3810-193 Aveiro, Portugal

**Keywords:** multiporphyrin, fullerene, supramolecular chemistry

## Abstract

A simple and straightforward synthesis of diporphyrins and pentaporphyrins is reported here. The supramolecular interactions of the new porphyrin derivatives with C_60_ and PyC_60_ (a pyridyl [60]fulleropyrrolidine) were evaluated by absorption and fluorescence titrations in toluene. While no measurable modifications of the absorption and fluorescence spectra were observed upon addition of C_60_ to the porphyrin derivatives, the addition of PyC_60_ to the corresponding mono-Zn(II) porphyrins resulted in the formation of Zn(porphyrin)–PyC_60_ coordination complexes and the binding constants were calculated. Results show that the four free-base porphyrin units in pentaporphyrin **6** have a significant contribution in the stabilization of the **6**–PyC_60_ complex. The crystal and molecular features of the pentaporphyrin Zn**5** were unveiled using single-crystal X-ray diffraction studies.

## 1. Introduction

Porphyrins are extremely versatile compounds from structural and chemical viewpoints. They display a rigid planar geometry, high stability, intense electronic absorption and emission, a small HOMO–LUMO energy gap, and flexible tunability of their optical and redox properties by changing the metal center [1]. Porphyrins can be efficiently modified at the *meso* or *beta* positions, or at the *meso*-(hetero)aryl substituents. Consequently, these compounds are one of the most attractive building blocks for the formation of covalent or supramolecular assemblies [2,3,4,5,6]. The design and synthesis of multiporphyrin arrays is an important research topic due to their potential application as artificial light-harvesting systems [7,8,9,10,11,12,13], functional materials (near infrared dyes, electron-conducting molecular wires, nonlinear optics, molecular recognition and sensing) [14,15,16,17], and also as agents for medical imaging and photodynamic therapy [18]. Several synthetic strategies have been developed to produce multiporphyrin oligomers, including directly *meso*–*meso*-, *meso*–*β*- and *β*–*β*-linked multiporphyrin systems, oligoporphyrins with fused π-systems and arrays bearing rigid or flexible spacers. In this context, different types of covalent or supramolecular porphyrin arrays with a large structural diversity [19,20,21,22], including linear [23,24,25], zig-zag [26], and dendritic arrays [27,28], tapes [29,30,31], belts [32], barrels [33], rings [34,35,36,37,38,39,40,41,42,43,44] and boxes [45] and balls [46], have been synthesized.

Fullerenes are another group of attractive building blocks, frequently used in association with porphyrins. In fact, architectures consisting of porphyrin derivatives (donors) and fullerenes (acceptors) are of particular interest, either as models for natural photosynthesis or for the conversion of light into electricity. Because of the unique three-dimensional structure of fullerenes, their low reduction potentials and small reorganization energy, providing the formation of long-lived charge separated states [47], porphyrin–fullerene photoactive systems have provided promising materials for photovoltaic applications [48,49,50,51,52,53,54]. In many cases, the photoactive system is a supramolecular complex formed by a multiporphyrin receptor and a fullerene guest [55,56,57,58,59,60,61,62,63]. Combining π–π interactions with hydrogen bonding [64] or axial coordination [65] is another strategy that results in the assembly of stable and robust architectures.

5,10,15,20-Tetrakis(pentafluorophenyl)porphyrin (**2**) reacts with a range of nucleophiles, namely amines, thiols, alcohols and phenols, leading to the formation of nucleophilic aromatic substitution products under mild conditions [66]. The *para*-fluorine atoms are selectively substituted by the nucleophile and mono- to tetra-substituted porphyrin derivatives may be obtained. We have reported the synthesis of multiporphyrin compounds via nucleophilic aromatic substitution of fluorine atoms in porphyrin **2** [20] or hexafluorobenzene [67,68]. Using a similar approach, herein we present a simple procedure for the preparation of diporphyrins and pentaporphyrins. The interaction of pentaporphyrins **5** and Zn**5** with C_60_ and pentaporphyrin **6** with 1-methyl-2-(4-pyridyl)[60]fullero[*c*]pyrrolidine (PyC_60_) is also discussed.

## 2. Results and Discussion

### 2.1. Synthesis and Characterization

**Scheme 1:**
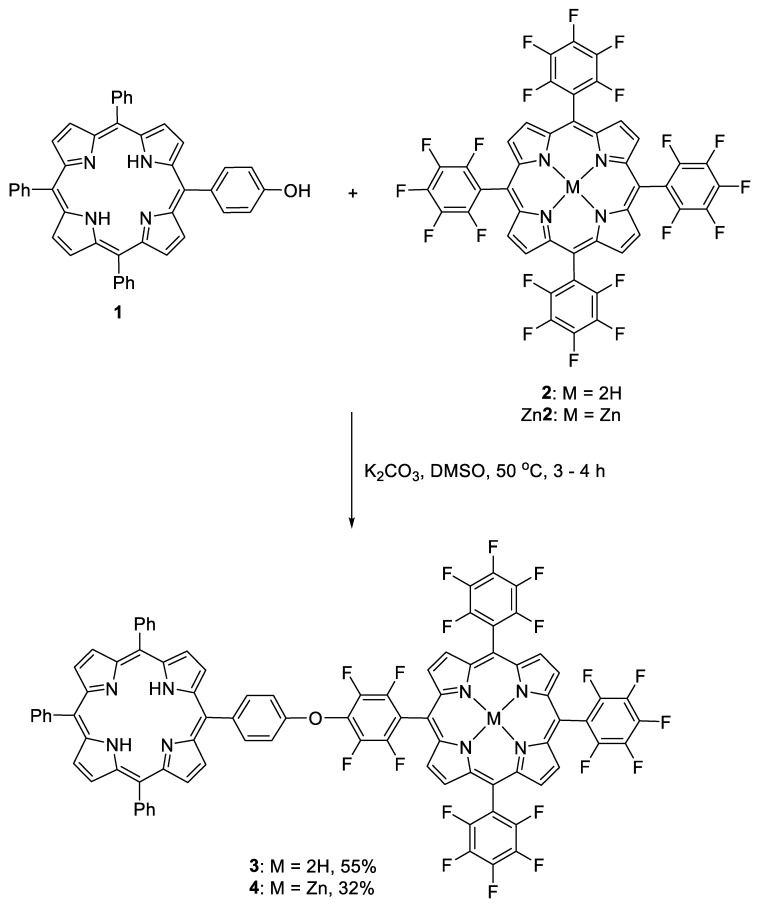
Synthesis of diporpyrins **3** and **4**.

**Figure 1:**
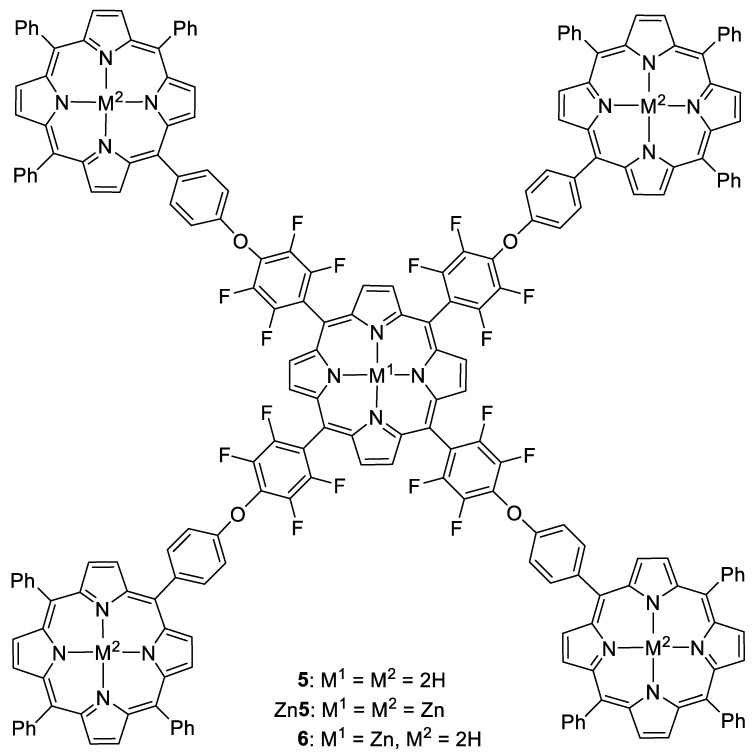
Molecular structures of pentaporphyrins **5**, Zn**5** and **6**.

We have shown that 5-(4-hydroxyphenyl)-10,15,20-triphenylporphyrin (**1**) reacts as a nucleophile with hexafluorobenzene or pentafluorophenyl groups to give multiporphyrin derivatives under very mild conditions [67]. As indicated above, porphyrin 2 reacts with nucleophiles to yield nucleophilic aromatic substitution products. Thus, it seemed obvious to us that we could access multiporphyrin compounds by reacting porphyrins **1** and **2** (Scheme 1 and Figure 1). In fact, the reaction of porphyrins **1** and **2** (in excess) afforded diporphyrin **3** in 55% yield; minor amounts of triporphyrin derivatives were also formed. Similarly, the reaction of porphyrin **1** with the Zn**2** complex, previously prepared by metalation of **2** with zinc acetate, produced diporphyrin **4** in 32% yield. When porphyrin **2** was treated with 5 equivalents of porphyrin **1**, the pentaporphyrin **5** (Figure 1) was obtained in 76% yield as the main product. Similarly, pentaporphyrin **6** (Figure 1) was obtained in 64% yield using **1** and Zn**2** as the starting porphyrins. Metalation of pentaporphyrin **5** with zinc acetate afforded Zn**5** in quantitative yield.

The structures of the new multiporphyrin compounds **3**–**6** and Zn**5** were confirmed by NMR, UV–Vis, and mass spectrometry. Comparing the ^1^H NMR spectra of diporphyrin **3** and pentaporphyrin **5**, the main differences are in the signals due to the resonances of the β-pyrrolic and NH protons. For diporphyrin **3**, the resonances of the β-pyrrolic protons of the fluorinated porphyrin appear as a singlet (at δ 8.93 ppm), and two doublets. However, due to the symmetry of pentaporphyrin **5**, only one singlet is observed (at 9.16 ppm) for the eight β-pyrrolic protons of the central porphyrin unit. Both spectra show two singlets assigned to the NH protons: −2.87 (2H) and −2.73 ppm (2H) for diporphyrin **3**, and −2.78 (8H) and −2.69 (2H) for pentaporphyrin **5**. The integration of those signals (1:1 or 1:4) agrees with the presence of two or five porphyrin units, for **3** and **5**, respectively. The ^1^H NMR spectra of diporphyrin **4** and pentaporphyrin **6** exhibit only one singlet at high field assigned to the NH protons (−2.74 ppm and −2.78 ppm, respectively). The ^19^F NMR spectra of the diporphyrins **3** and **4** and the pentaporphyrins **5** and **6** further confirm the proposed structures, particularly the number of the *para*-fluorine atoms substituted by porphyrin units. The ^19^F NMR spectra of diporphyrins **3** and **4** show a multiplet between δ −174.82 and −174.65 ppm for diporphyrin **3**, and between δ −175.03 and −174.85 ppm for diporphyrin **4**, assigned to the three *para*-fluorine atoms. On the other hand, the absence of signals due to the *para*-fluorine atoms in the ^19^F NMR spectra of pentaporphyrins **5** and **6** confirm the tetra-substitution. The mass spectra of diporphyrins **3** and **4** show the expected [M + H]^+^ ions (*m*/*z* = 1585.3 for diporphyrin **3** and *m*/*z* = 1647.3 for diporphyrin **4**). The HRMS (ESI) of diporphyrin **3** also shows a peak at *m*/*z* = 793.2 corresponding to the [M + 2H]^2+^ ion, which results from multiple protonation of the molecule (Figure S4). The MALDI-TOF mass spectra of pentaporphyrins **5** and **6** reveal the peaks corresponding to the monoisotopic masses of the [M + H]^+^ ions (*m*/*z* = 3416.0 for pentaporphyrin **5** and *m*/*z* = 3477.9 for pentaporphyrin **6**). However, the HRMS (ESI) of pentaporphyrin **5** shows only the peaks corresponding to the [M + 2H]^2+^ and [M + 3H]^3+^.

**Figure 2:**
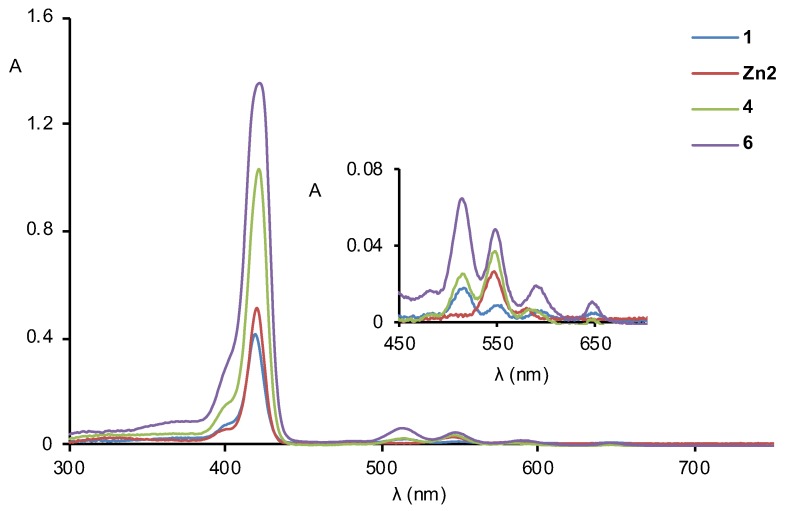
Absorption spectra of **1**, Zn**2**, **4**, and **6** in toluene. All compounds were used with a concentration of 1.0 μM. The inset shows an expansion of the Q bands.

Comparing the UV–Vis spectra of diporphyrins and pentaporphyrins with those of their porphyrin precursors (Figure 2), the sole difference is the intensity of the Soret and Q bands, ultimately confirming that there is no conjugation between the porphyrin units.

**Figure 3:**
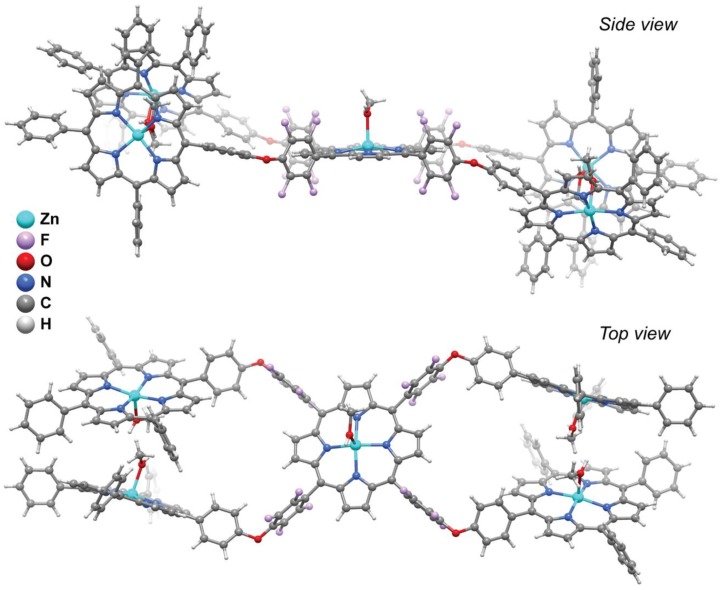
Schematic representation of the pentaporphyrin molecular unit present in the crystal structure of Zn**5**·*Solvent*.

Pentaporphyrin Zn**5** was isolated as large red single-crystals through recrystallization from a mixture of chloroform and methanol (among other solvents in minor quantity). Figure 3 depicts the centrosymmetric molecular unit present in the crystal structure of Zn**5**·*Solvent* (side and top views), clearly reflecting the high level of conformational flexibility of the large molecule. The three crystallographically independent Zn^2+^ metal centers exhibit identical coordination environments which strongly resemble a distorted square pyramid: while the basal plane is formed by the four nitrogen atoms arising from the porphyrinic core [Zn–N distances ranging from 2.040(3) to 2.089(4) Å (for the three environments)], the apical position is always occupied by a coordinated methanol molecule with the Zn–O_methanol_ distance being found in the 2.118(5)–2.197(5) Å range. As also depicted in Figure 3, the four peripheral porphyrin molecules adopt a conformation so they are close together, mutually interacting via weak supramolecular contacts most certainly also involving the solvent molecules of crystallization (not shown).

### 2.2. Formation of Porphyrin–Fullerene Complexes

**Figure 4:**
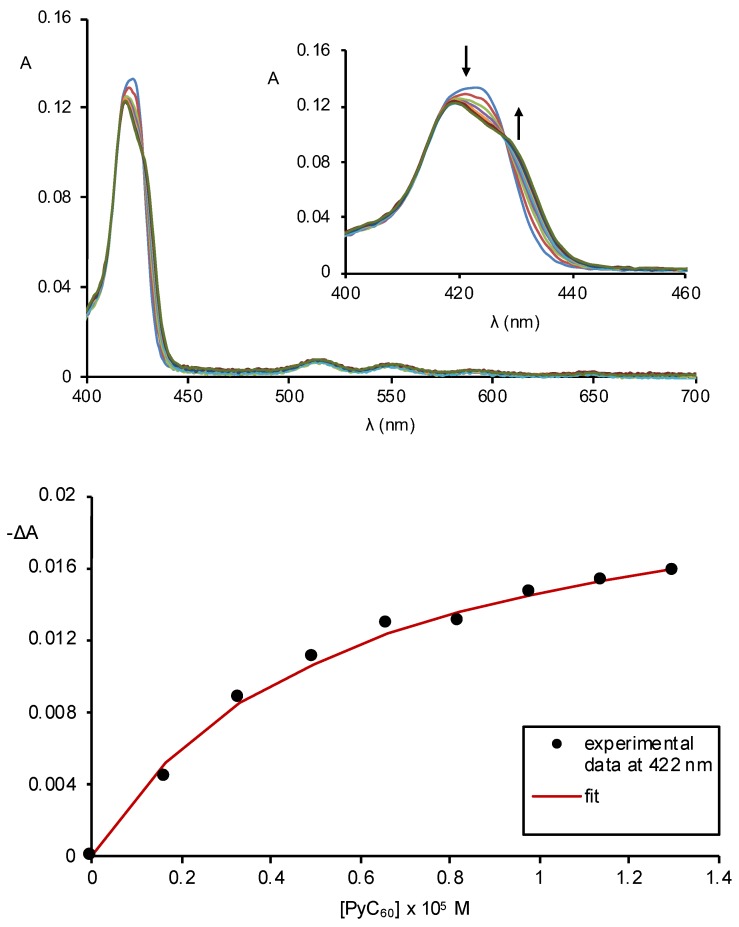
Absorption spectra of **6** (1.0 × 10^−7^ M) upon addition of PyC_60_ (0–113 equiv.) in toluene at 23 °C (**upper part**) and experimental data at 422 nm fitted to a non-linear 1:1 binding model (**lower part**).

**Figure 5:**
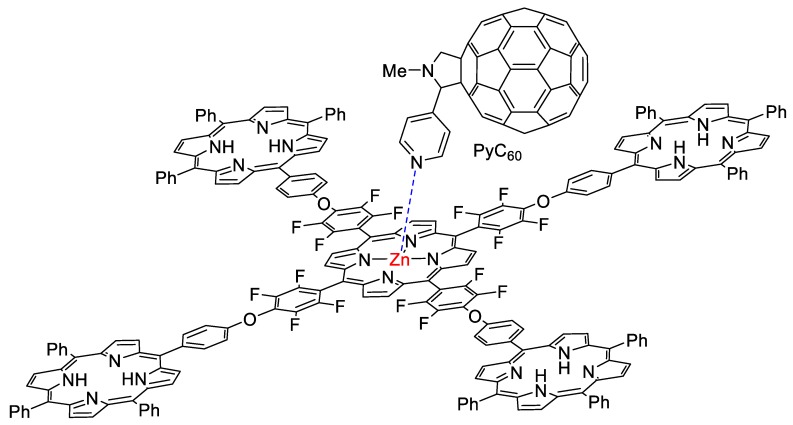
Representation of the coordination complex **6**–PyC_60_.

The binding capabilities of pentaporphyrins **5** and Zn**5** to form complexes with pristine C_60_ was investigated by absorption and fluorescence titrations in toluene. Surprisingly, the addition of a toluene solution of C_60_ to pentaporphyrins **5** and Zn**5** did not induce a quantifiable alteration in their absorption and fluorescence spectra. This indicates that the eventual π-π interactions established between the host and C_60_ are too weak, in solution, to induce spectral modifications. However, the titration of pentaporphyrin **6** with PyC_60_ revealed a markedly distinct behavior. The addition of PyC_60_ to a solution of **6** in toluene induced a decrease of the Soret band (at 422 nm) along with the appearance of an isosbestic point at 428 nm (Figure 4). This suggests the formation of a **6**–PyC_60_ complex, presumably via axial coordination of the pyridyl group to the zinc porphyrin (Figure 5), as reported in similar systems [56,68,69,70,71,72].

To evaluate the contribution of the four free porphyrin units in the stabilization of the **6**–PyC_60_ complex, the precursor Zn**2** and the diporphyrin **4** were also titrated with PyC_60_. As expected, the addition of PyC_60_ to Zn**2** induced a red shift of the Soret and Q bands with the formation of an isosbestic point at 423 nm (Figure S14 in the ESI), probably as a result of axial coordination of the pyridyl group of PyC_60_ to Zn**2**. D’Souza already observed the complex formation between porphyrin **2** and PyC_60_ in dichloromethane [73]. Similar behavior was observed when a solution of diporphyrin **4** in toluene was titrated with PyC_60_. The incremental addition of PyC_60_ caused a decrease and a red shift of the Soret with the formation of an isosbestic point at 425 nm (Figure S16 in the ESI). A red shift was observed in the Q bands, which is most likely a consequence of coordination complex formation. As discussed below, the binding constant for the **6**–PyC_60_ complex is ca. 5.8 times higher than the binding constant for the Zn**2**–PyC_60_ complex, which confirms that the four free-base porphyrin units in pentaporphyrin **6** have a significant contribution in the stabilization of the **6**–PyC_60_ complex. The role of the four peripheral porphyrin units in stabilizing the **6**–PyC_60_ complex may be compared to the synergic effect observed in C_60_ and C_70_ hosting behavior of corannulene- and pyrene-substituted porphyrins [74,75,76].

**Figure 6:**
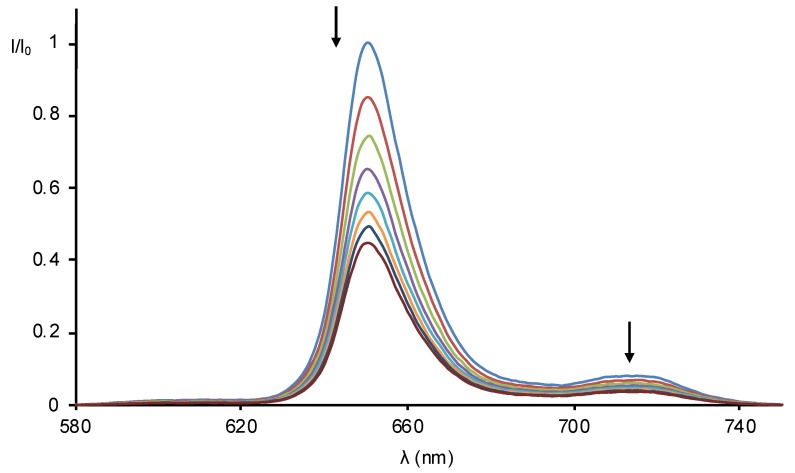

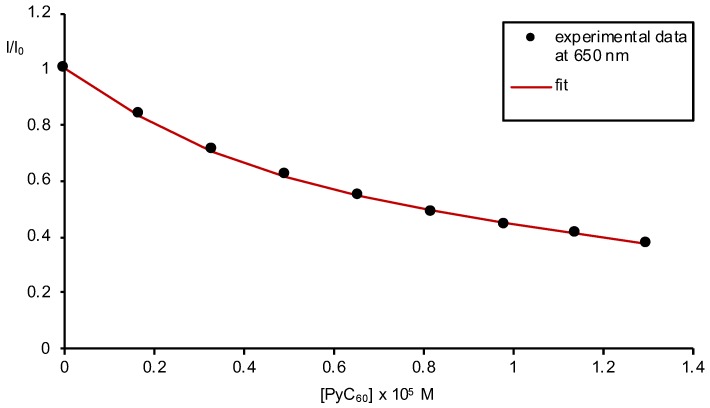
Fluorescence spectra (λ_exc_ = 428 nm) of **6** (1.0 × 10^−7^ M) upon addition of PyC_60_ (0–113 equiv.) in toluene at 23 °C (**upper part**) and experimental data at 650 nm fitted to a non-linear 1:1 binding model (**lower part**).

The steady state fluorescence spectrum of pentaporphyrin **6** displays two bands centered at 650 and 715 nm (Figure 6). Upon addition of PyC_60_ to a solution of **6** in toluene, the fluorescence intensity decreased significantly when exciting at 428 nm, as depicted in Figure 6. These changes are fully consistent with strong interactions between the photoexcited **6** and PyC_60_ in the **6**–PyC_60_ complex [68,70,73]. The fluorescence quenching of the precursor Zn**2** and diporphyrin **4** upon addition of PyC_60_ was also observed (Figures S15 and S17 in the ESI).

The binding constants (*K*) were obtained from the absorption and fluorescence spectral data by using a non-linear 1:1 binding model (Figure 4 and Figure 6) and are listed in Table 1. The average values for the binding constants (*K*_av_) increase in the order Zn**2** < **4** < **6**. In particular, the *K*_av_ value of the **6**–PyC_60_ complex (*K*_av_ = 1.53 × 10^5^ M^−1^) was found to be ca. 5.8 times higher than the *K*_av_ for the Zn**2**–PyC_60_ complex (*K*_av_ = 2.66 × 10^4^ M^−1^). As discussed above, the enhanced binding ability of the pentaporphyrin **6** arises presumably from the synergic effect of the π–π interactions between the fullerene unit and the four free-base porphyrin units.

## 3. Materials and Methods

### 3.1. Chemicals and Instrumentation

^1^H, ^13^C and ^19^F NMR spectra were recorded on Bruker AVANCE 300 or Bruker AVANCE 500 spectrometers. CDCl_3_ was use as solvent and tetramethylsilane (TMS) as internal reference. The chemical shifts are expressed in δ (ppm) and the coupling constants (*J*) in hertz (Hz). UV–Vis spectra were recorded on a Shimadzu UV-2501PC spectrophotometer using toluene or CHCl_3_ as solvent. UV−vis absorption spectral wavelengths (λ) are reported in nanometers (nm), and molar absorption coefficients (ε) are reported in M^−1^ cm^−1^. Fluorescence emission spectra were recorded on a JASCO FP-8300 spectrofluorometer. Mass spectra were recorded using a MALDI TOF/TOF 4800 Applied Biosystems MDS Sciex mass spectrometer, CHCl_3_ as solvent and 3-nitrobenzyl alcohol (NBA) as matrix. High-resolution mass spectra (HRMS) were recorded on a Bruker Apex-Qe FTICR mass spectrometer or on a LTQ Orbitrap XL mass spectrometer using CHCl_3_ as solvent. Melting points were measured on a Büchi B-540 apparatus and are uncorrected. Column chromatography was carried out using silica gel (Merck, 35-70 mesh). Analytical TLC was carried out on precoated sheets with silica gel (Merck 60, 0.2 mm thick). Solvents were purified or dried according to the literature procedures [77]. Compounds **1** [78], **2** [78] and PyC_60_ [79] were prepared according to published procedures.

### 3.2. Synthesis

#### 3.2.1. 5,10,15,20-Tetrakis(pentafluorophenyl)porphyrinatozinc(II) (Zn**2**)

Prepared as in [73]. Zinc acetate (28.2 mg, 0.154 mmol) was added to a solution of **2** (50.0 mg, 51.3 μmol) in chloroform/methanol (2:1) and the resulting mixture was stirred at 60 °C for 15 min. After cooling down to ambient temperature, the reaction mixture was washed with distilled water. The organic phase was dried (Na_2_SO_4_) and the solvent was evaporated under reduced pressure. The Zn**2** complex was obtained in quantitative yield. UV–Vis (toluene): λ_max_ (log ε) 421 (5.5), 546 (4.2), 581 (3.7) nm. MS (MALDI-TOF): *m*/*z* 1036.0 [M]^+^.

#### 3.2.2. Diporphyrin **3**

A solution of **1** (20.0 mg, 31.7 μmol), **2** (92.7 mg, 95.1 μmol) and potassium carbonate (13.1 mg, 95.1 μmol) in dry DMSO (3 mL) was stirred under a nitrogen atmosphere at 50 °C for 3 h. After cooling to ambient temperature, the porphyrinic compounds were precipitated with an aqueous solution of citric acid, filtered and washed with water. The solid was dissolved in dichloromethane and then washed with water. The organic phase was dried (Na_2_SO_4_) and the solvent was evaporated under reduced pressure. The residue was purified by column chromatography on silica gel using a gradient of dichloromethane/hexane. The first fraction was identified as the unreacted porphyrin **2**. The following fraction afforded diporphyrin **3** (28 mg, 55% yield) after crystallization from dichloromethane/methanol. mp > 300 °C. ^1^H NMR (CDCl_3_, 300 MHz): δ, ppm −2.87 (s, 2H, NH of moiety **1**), −2.73 (s, 2H, NH of moiety **2**), 7.71–7.82 (m, 11H, C_6_H_4_-*m*-H and Ph-*m*,*p*-H), 8.22–8.26 (m, 6H, Ph-*o*-H), 8.37 (d, *J* = 8.6 Hz, 2H, C_6_H_4_-*o*-H), 8.87 (s, 4H, β-H of moiety **1**), 8.91–8.97 (3 overlapped d, 6H, β-H), 8.93 (s, 4H, β-H of moiety **2**), 9.09 (d, *J* = 4.8 Hz, 2H, β-H of moiety **2**). ^13^C NMR (CDCl_3_, 126 MHz): δ, ppm 103.6, 104.4, 114.4, 118.6, 120.3, 120.4, 126.7, 127.8, 131.1 (br s, β-C), 134.6, 135.9, 138.4, 142.1, 157.2. ^19^F NMR (CDCl_3_, 282 MHz): δ, ppm −184.83 (dt, *J* = 23.2 and 7.6 Hz, 6F, C_6_F_5_-*m*-F), −176.86 (dd, *J* = 23.1 and 9.3 Hz, 2F, C_6_F_4_-*m*-F), −174.82 to −174.65 (m, 3F, C_6_F_5_-*p*-F), −160.40 (dd, *J* = 23.1 and 9.3 Hz, 2F, C_6_F_4_-*o*-F), −160.00 (dd, *J* = 23.2 and 7.6 Hz, 6F, C_6_F_5_-*o*-F). UV–Vis (CHCl_3_): λ_max_ (log ε) 418 (5.8), 510 (4.3), 548 (4.2), 586 (3.8), 644 (3.7) nm. MS (MALDI-TOF): *m*/*z* 1585.3 [M + H]^+^. MS (HRESI): *m*/*z* calcd. for C_88_H_40_F_19_N_8_O [M + H]^+^ 1585.3016, found 1585.3037, calcd. for C_88_H_41_F_19_N_8_O [M + 2H]^2+^ 793.1545, found 793.1534.

#### 3.2.3. Diporphyrin **4**

A solution of **1** (20.0 mg, 31.7 μmol), Zn**2** (84.2 mg, 95.1 μmol) and potassium carbonate (13.1 mg, 95.1 μmol) in dry DMSO (3 mL) was stirred under a nitrogen atmosphere at 50 ºC for 4 h. The workup procedures were the same as previously described for diporphyrin **3**. The residue was purified by column chromatography on silica gel using a gradient of dichloromethane/hexane. The first fraction was identified as the starting porphyrin Zn**2**. The following fraction afforded diporphyrin **4** (23 mg, 32% yield) after crystallization from dichloromethane/methanol. mp > 300 ºC. ^1^H NMR (CDCl_3_, 300 MHz): δ, ppm −2.74 (s, 2H, NH), 7.72–7.83 (m, 11H, C_6_H_4_-*m*-H and Ph-*m*,*p*-H), 8.23–8.27 (m, 6H, Ph-*o*-H), 8.38 (d, *J* = 8.5 Hz, 2H, C_6_H_4_-*o*-H), 8.88 (s, 4H, β-H of moiety **1**), 8.93 and 8.98 (AB, *J* = 4.8 Hz, β-H of moiety **1**), 9.01 (s, 4H, β-H of moiety Zn**2**), 9.04 and 9.17 (AB, *J* = 4.7 Hz, 4H, β-H of moiety Zn**2**). ^19^F NMR (CDCl_3_, 282 MHz): δ, ppm −184.75 (dt, *J* = 23.6 and 8.0 Hz, 6F, C_6_F_5_-*m*-F), −176.77 (dd, *J* = 23.4 and 9.4 Hz, 2F, C_6_F_4_-*m*-F), −175.03 to −174.85 (m, 3F, C_6_F_5_-*p*-F), −160.21 (dd, *J* = 23.4 and 9.4 Hz, 2F, C_6_F_4_-*o*-F), −159.85 (dd, *J* = 23.6 and 8.0 Hz, 6F, C_6_F_5_-*o*-F). UV–Vis (toluene): λ_max_ (log ε) 421 (5,8), 513 (4,3), 546 (4,4), 584 (3,9), 646 (3,7) nm. MS (MALDI-TOF): *m*/*z* 1647.3 [M + H]^+^. MS (HRESI): *m*/*z* calcd. for C_88_H_38_F_19_N_8_OZn [M + H]^+^ 1647.2078, found 1647.2110.

#### 3.2.4. Pentaporphyrin **5**

A solution of **2** (10.0 mg, 10.2 μmol), **1** (32.4 mg, 51.3 μmol) and potassium carbonate (17.0 mg, 0.123 mmol) in dry DMSO (1 mL) was stirred under a nitrogen atmosphere at 80 °C for 2 h. After cooling to ambient temperature, the porphyrinic compounds were precipitated with an aqueous solution of citric acid, filtered and washed with water. The solid was dissolved in dichloromethane and then washed with water. The organic phase was dried (Na_2_SO_4_) and the solvent was evaporated under reduced pressure. The residue was purified by column chromatography on silica gel using dichloromethane/hexane (2:1) as the eluent. A major faction was eluted and then the unreacted porphyrin **1** was recovered. The major fraction afforded pentaporphyrin **5** (27 mg, 76% yield) after crystallization from dichloromethane/methanol. mp > 300 °C. ^1^H NMR (CDCl_3_, 300 MHz): δ, ppm −2.78 (s, 8H, NH of moiety **1**), −2.69 (s, 2H, NH of moiety **2**), 7.63–7.78 (m, 44H, C_6_H_4_-*m*-H and Ph-*m*,*p*-H), 8.15 (dd, *J* = 7.5 and 1.8 Hz, 16H, 10,20-Ph-*o*-H), 8.22 (dd, *J* = 7.3 and 1.7 Hz, 8H, 15-Ph-*o*-H), 8.33 (d, *J* = 8.7 Hz, 8H, C_6_H_4_-*o*-H), 8.81 (d, *J* = 6 Hz, 8H, β-H of moiety **1**), 8.83–8.86 (m, 16H, β-H of moiety **1**), 8.81 (d, *J* = 6 Hz, 8H, β-H of moiety **1**), 9.16 (s, 8H, β-H of moiety **2**). ^13^C NMR (CDCl_3_, 75 MHz): δ, ppm 104.3, 114.3, 116.7, 118.6, 120.2, 120.3, 126.6, 126.7, 127.7, 129.8–132.4 (β-C), 134.5, 135.9, 138.3, 142.0, 142.1, 157.1. ^19^F NMR (CDCl_3_, 282 MHz): δ, ppm -176.92 (dd, *J* = 23.1 and 9.2 Hz, 8F, C_6_F_4_-*m*-F), −160.33 (dd, *J* = 23.1 and 9.2 Hz, 8F, C_6_F_4_-*o*-F). UV–Vis (toluene): λ_max_ (log ε) 420 (6.2), 513 (5.0), 547 (4.6), 590 (4.5), 648 (4.3) nm. MS (MALDI-TOF): *m*/*z* 3416.0 [M + H]^+^. MS (HRESI): *m*/*z* calcd. for C_220_H_128_F_16_N_20_O_4_ [M + 2H]^2+^ 1708.5081, found 1708.5129, calcd. for C_220_H_129_F_16_N_20_O_4_ [M + 3H]^3+^ 1139.3411, found 1139.3427.

#### 3.2.5. Pentaporphyrin Zn**5**

Zinc acetate (16.1 mg, 87.8 mmol) was added to a solution of **5** (20.0 mg, 5.85 μmol) in chloroform/methanol (2:1) and the resulting mixture was stirred at 60 °C for 1 h. After cooling down to ambient temperature, the reaction mixture was washed with distilled water. The organic phase was dried (Na_2_SO_4_) and the solvent was evaporated under reduced pressure. The Zn**5** complex was obtained in quantitative yield. ^1^H NMR (CDCl_3_, 300 MHz): δ, ppm 7.57–7.74 (m, 44H, C_6_H_4_-*m*-H and Ph-*m*,*p*-H), 8.09–8.18 (m, 24H, Ph-*o*-H), 8.28 (d, *J* = 8.6 Hz, 8H, C_6_H_4_-*o*-H), 8.80–8.86 (m, 24H, β-H of moiety **1**), 8.92 (d, *J* = 4.7 Hz, 8H, β-H of moiety Zn**1**), 9.13 (s, 8H, β-H of moiety Zn**2**). UV–Vis (toluene): λ_max_ (log ε) 424 (6.1), 548 (4.9), 588 (4.4) nm. MS (MALDI-TOF): *m*/*z* 3734,3 [M]^+**·**^.

#### 3.2.6. Pentaporphyrin 6

A solution of Zn**2** (10.0 mg, 11.3 μmol), **1** (35.6 mg, 56.4 μmol) and potassium carbonate (18.7 mg, 0.135 mmol) in dry DMSO (1 mL) was stirred under a nitrogen atmosphere at 80 ºC for 2 h. The workup procedures were the same as described for pentaporphyrin **5**. The residue was purified by column chromatography on silica gel using dichloromethane/hexane (2:1) as the eluent. A major faction was eluted and then unreacted porphyrin **1** was recovered. The major fraction afforded pentaporphyrin **6** (25 mg, 64% yield) after crystallization from dichloromethane/hexane. mp > 300 ºC. ^1^H NMR (CDCl_3_, 300 MHz): δ, ppm -2.78 (s, 8H, NH), 7.60–7.78 (m, 44H, 5-C_6_H_4_-*m*-H and 10,15,20-Ph-*m*,*p*-H), 8.15 (dd, *J* = 7.4 and 1.8 Hz, 16H, 10,20-Ph-*o*-H), 8.22 (dd, *J* = 7.4 and 1.7 Hz, 8H, 15-Ph-*o*-H), 8.34 (d, *J* = 8.5 Hz, 8H, 5-C_6_H_4_-*o*-H), 8.80–8.86 (m, 24H, β-H of moiety **1**), 8.93 (d, *J* = 4.8 Hz, 8H, β-H of moiety **1**), 9.25 (s, 8H, β-H of moiety Zn**2**). ^13^C NMR (CDCl_3_, 126 MHz): δ, ppm 105.0, 114.3, 117.4, 117.6, 117.7, 118.7, 120.28, 120.34, 126.65, 126.73, 127.7, 127.8, 129.8–132.3 (β-C), 132.4, 134.5, 134.6, 135.9, 138.3, 140.6, 140.8, 142.0, 142.1, 142.7, 142.8, 146.0, 148.1, 150.4, 157.2. ^19^F NMR (CDCl_3_, 282 MHz): δ, ppm -176.81 (dd, *J* = 23.4 and 9.2 Hz, 8F, C_6_F_4_-*m*-F), -160.14 (dd, *J* = 23.4 and 9.2 Hz, 8F, C_6_F_4_-*o*-F). UV–Vis (toluene): λ_max_ (log ε) 422 (6.1), 513 (4.8), 549 (4.7), 589 (4.4), 645 (4.3) nm. MS (MALDI-TOF): *m/z* 3477.9 [M + H]^+^.

### 3.3. Absorption and Fluorescence Titrations

The UV–Vis and fluorescence titrations were carried out using a stock solution of each compound in toluene in a quartz cuvette (1 cm path length). A PyC_60_ stock solution was added in aliquots and the spectra were recorded after each addition. Absorption spectra were corrected for the contribution of the added PyC_60_. In the fluorescence measurements the sample was excited at the isosbestic point. Measurements were repeated 2–3 times and found to be reproducible within a margin of error of ca. 15%. All measurements were performed at 23 °C.

The binding constants (*K*) were assessed from the UV–Vis and fluorescence titrations. The variation of the absorbance at the Soret band caused by consecutive additions of PyC_60_ was used to determine the binding constants. The spectral changes at the Soret band were fit using non-linear least-squares procedures using the following equation for a 1:1 binding:

(1) $$A=\frac{\varepsilon_{\mathrm{Por}}\left[\mathrm{Por}\right]+\varepsilon_{\mathrm{Por}-\mathrm{Ful}}K\left[\mathrm{Por}\right]\left[\mathrm{Ful}\right]}{1+K\left[\mathrm{Ful}\right]}$$

in which A is the absorbance, [Por] is the total concentration of the porphyrin derivative, [Ful] is the concentration of PyC_60_, ε_Por_ and ε_Por-Ful_ are constants associated with the molar extinction coefficients of the porphyrin derivative and the complex formed, respectively.

In a similar way, the spectral changes at maximum fluorescence intensity were fit using non-linear least-squares procedures using the following equation for 1:1 binding:

(2) $$I=\frac{\Phi_{\mathrm{Por}}\left[\mathrm{Por}\right]+\Phi_{\mathrm{Por}-\mathrm{Ful}}K\left[\mathrm{Por}\right]\left[\mathrm{Ful}\right]}{1+K\left[\mathrm{Ful}\right]}$$

where I is the fluorescence intensity, Φ_Por_ and Φ_Por-Ful_ are constants associated with the emission quantum yields of the porphyrin derivative and of the complex formed, respectively [80].

### 3.4. Single-Crystal X-Ray Diffraction Studies of Zn**5**·Solvent

Single crystals of compound Zn**5**·*Solvent* were manually harvested from the crystallization vial and immersed in highly viscous FOMBLIN Y perfluoropolyether vacuum oil (LVAC 140/13, Sigma-Aldrich) to avoid degradation caused by the evaporation of the solvent [81]. Crystals were mounted on Hampton Research CryoLoops with the help of a Stemi 2000 stereomicroscope equipped with Carl Zeiss lenses. X-ray diffraction data were collected at 179(2) K on a Bruker D8 QUEST equipped with Mo Kα sealed tube (λ = 0.71073 Å), a multilayer TRIUMPH X-ray mirror, a PHOTON 100 CMOS detector, and an Oxford Instruments Cryostrem 700+ Series low temperature device. Diffraction images were processed using the software package SAINT+ [82], and data were corrected for absorption by the multiscan semi-empirical method implemented in SADABS [83]. The structure was solved using the algorithm implemented in SHELXT-2014 [84], which allowed for the immediate location of almost all of the heaviest atoms composing the molecular unit. The remaining missing and misplaced non-hydrogen atoms were located from difference Fourier maps calculated from successive full-matrix least-squares refinement cycles on *F*^2^ using the latest SHELXL from the 2017/1 release [85]. All structural refinements were performed using the graphical interface ShelXle [86].

The crystal diffracted poorly throughout the entire angular range, this despite a long acquisition time per frame (*ca*. 1 min) and the use of a wide frame strategy (0.5° ϕ scans). Indeed, below 1.0 Å of resolution the mean *I*/*σ* drops below 5.0 with the *R*_merge_ value being concomitantly high (for example, for a resolution of 1.20 Å the *R*_merge_ is already above 10%). The poor quality of the overall diffraction has important consequences in the modeled structure, giving rise to a number of alerts in PLATON [87,88]. Nevertheless, structure solution immediately showed the presence of the most important chemical moiety in the crystal structure.

Besides the pentaporphyrin Zn**5** derivative, the crystal structure contains one large void and two smaller ones (total volume of about 2446 Å^3^ as estimated by PLATON) [87,88] centred at (0.385 0.3305 0.000), (0.263 −0.819 0.417), and (0.736 0.181 0.583), which most likely contain highly disordered solvent molecules. From difference Fourier maps it was possible to discern the presence of a considerable smeared-out electron density in these locations. Nevertheless, several attempts to locate and model solvent molecules proved to be unproductive. The original data set was treated using the *SQUEEZE* [89] subroutines implemented in PLATON [87,88] in order to remove the contribution of these highly disordered molecules. It was estimated that the aforementioned cavities would contain a total of ca. 632 electrons. The calculated solvent-free reflection list was used for subsequent structural refinements that converged to the solvent-free structure reported in this manuscript and having the reliability factors listed below.

The asymmetric unit is composed of half of the pentaporphyrin in which the metallic centres are coordinated to a methanol molecule. These three crystallographically independent methanol molecules were found to be severely affected by positional disorder and were included in the final structural model with the distances restrained to common (refineable) distances and with isotropic displacement parameters for the non-hydrogen atoms, so as to ensure chemically reasonable geometries for these moieties.

Hydrogen atoms bound to carbon were placed at their idealized positions using appropriate *HFIX* instructions in SHELXL: *43* (aromatic carbon atoms and coordinated hydroxyl groups) and *137* (for all methyl groups). These hydrogen atoms were included in subsequent refinement cycles with isotropic thermal displacements parameters (*U*_iso_) fixed at 1.2 (for the former family of hydrogen atoms) or 1.5×*U*_eq_ (solely for those associated with the methyl groups) of the parent atoms.

The last difference Fourier map synthesis showed the highest peak (1.947 eÅ^−3^) and the deepest hole (-1.354 eÅ^−3^) located at 0.39 and 0.07 Å from H2M and C2M (associated with the coordinated methanol molecule), respectively. Structural drawings have been created using the software package Crystal Impact Diamond [90].

*Crystal data for* Zn**5**·*Solvent* (SQUEEZE Data): C_225_H_136_F_16_N_20_O_9_Zn_5_, *M* = 3894.38, triclinic, space group *P*ī, *Z* = 1, *a* = 12.995(2) Å, *b* = 15.034(3) Å, *c* = 34.607(7) Å, *α* = 78.338(10)°, *β* = 81.133(9)°, *γ* = 79.449(10)°, *V* = 6461(2) Å^3^, *μ*(Mo-Kα) = 0.518 mm^−1^, *D*_c_ = 1.001 g cm^−3^, red block with crystal size of 0.20 × 0.10 × 0.05 mm^3^. Of a total of 127,498 reflections collected, 23,500 were independent (*R*_int_ = 0.0640). Final *R*1 = 0.0769 [*I* > 2*σ(I)*] and *wR*2 = 0.2353 (all data). Data completeness to theta = 25.24°, 99.5%.

Crystallographic data (including structure factors) for the crystal structure of compound Zn**5**·*Solvent* have been deposited with the Cambridge Crystallographic Data Centre as supplementary publication No. CCDC-1825479. Copies of the data can be obtained free of charge on application to CCDC, 12 Union Road, Cambridge CB2 2EZ, UK. FAX: (+44) 1223 336033. E-mail: deposit@ccdc.cam.ac.uk.

## 4. Conclusions

The reaction of 5-(4-hydroxyphenyl)-10,15,20-triphenylporphyrin (**1**) with 5,10,15,20-tetrakis(pentafluorophenyl)porphyrin (**2**), under mild conditions affords a diporphyrin (**3**) or a pentaporphyrin (**5**) selectively and in good yields. Similarly, the reaction of **1** with Zn**2** leads to the nono-Zn(II) diporphyrin **4** or to the mono-Zn(II) pentaporphyrin **6**.

Absorption and fluorescence titrations of the new porphyrin derivatives with C_60_ in toluene did not show the formation of supramolecular complexes. However, titrations with PyC_60_ lead to the formation of Zn(porphyrin)–PyC_60_ coordination complexes and the corresponding binding constants were calculated. Comparing the binding constants for the Zn**2**–PyC_60_ complex (*K*_av_ = 2.66 × 10^4^ M^−1^) and the **6**–PyC_60_ complex (*K*_av_ = 1.53 × 10^5^ M^−1^), it is evident that the four free-base porphyrin units in pentaporphyrin **6** have a significant contribution in the stabilization of the **6**–PyC_60_ complex.

The method reported here for the synthesis of pentaporphyrins **5** and **6** may be useful to decorate a central porphyrin with one to four structural units with specific functions. Considering that porphyrin–fullerene systems are potentially useful for photovoltaic applications, this method may be used to decorate a porphyrin with other photoactive units or substituents able to make strong binding interactions with fullerenes (pyrene of corannulene units, for instance).

## Figures and Tables

**Table 1:** Binding constants (*K*, M^−1^) calculated by absorption and fluorescence measurements for Zn**2**–PyC_60_, **4**–PyC_60_ and **6**–PyC_60_ complexes in toluene at 23 °C.

Complex	*K* (M^−1^)		*K*_av_ (M^−1^) *^a^*
	Absorption	Fluorescence	
Zn2–PyC_60_	1.75 × 10^4^	3.57 × 10^4^	2.66 × 10^4^
4–PyC_60_	5.04 × 10^4^	4.59 × 10^4^	4.82 × 10^4^
6–PyC_60_	1.77 × 10^5^	1.28 × 10^5^	1.53 × 10^5^

*^a^ K*_av_ = average of the two calculated *K* values.

## Supplementary Materials

The following are available online: ^1^H, ^13^C and ^19^F NMR spectra and absorption and fluorescence titrations with PyC_60_.
