# Supplementary material for: A Convenient Synthesis of Pentaporphyrins and Supramolecular Complexes with a Fulleropyrrolidine
Source: Molecules. 2019 Sep 1;24(17):3177. doi: 10.3390/molecules24173177 (PMC6749455; doi:10.3390/molecules24173177)
Supplement: Supplementary file 1 [file molecules-24-03177-s001.pdf]

Supporting Information

A convenient synthesis of pentaporphyrins and supramolecular complexes with a fulleropyrrolidine

Joana I. T. Costa,[a] Andreia S. F. Farinha,[b] Filipe A. Almeida Paz,[c] and Augusto C. Tomé [a,]*

[a] *QOPNA and LAQV-REQUIMTE, Department of Chemistry, University of Aveiro, 3810-193 Aveiro, Portugal*

[b]*King Abdullah University of Science and Technology (KAUST), Water Desalination and Reuse Center (WDRC), Division of Biological and Environmental Sciences (BESE), Thuwal, Saudi Arabia*

[c] *Department of Chemistry, CICECO – Aveiro Institute of Materials, University of Aveiro, 3810-193 Aveiro, Portugal*

INDEX

	Page
**1. NMR and mass spectra**	**2**
**2. Absorption and fluorescence titrations with PyC_{60}**	**9**

1. NMR and mass spectra

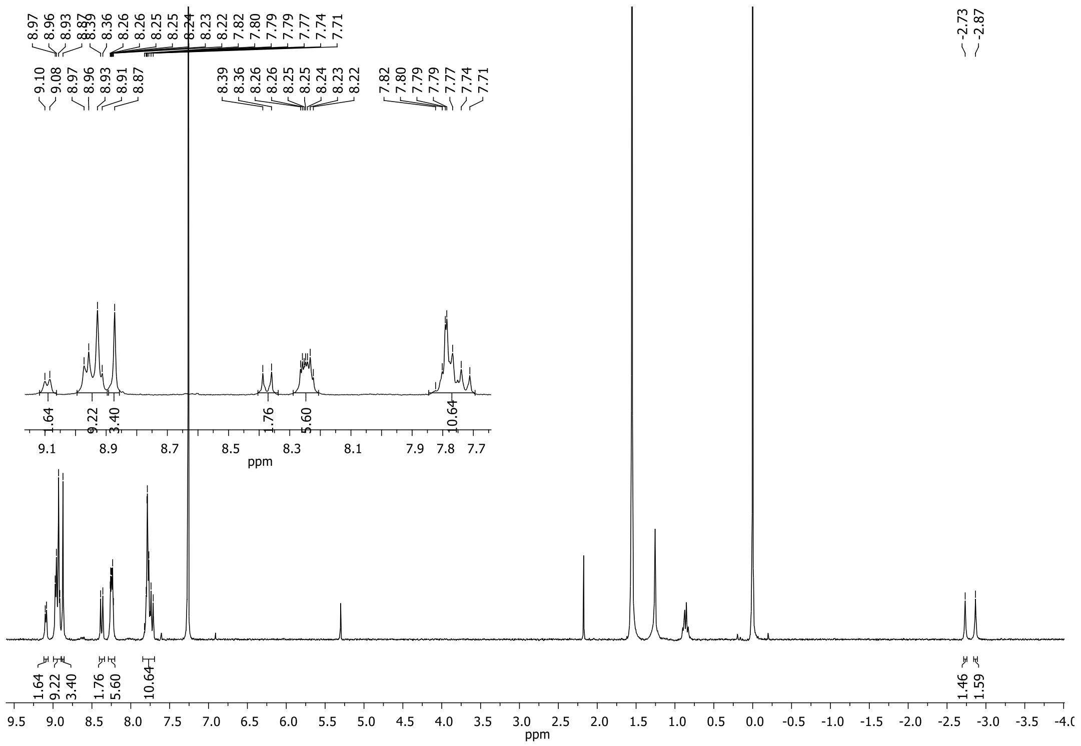

Figure S1: ^{1}H NMR spectrum of diporphyrin **3** (in $CDCl_3$).

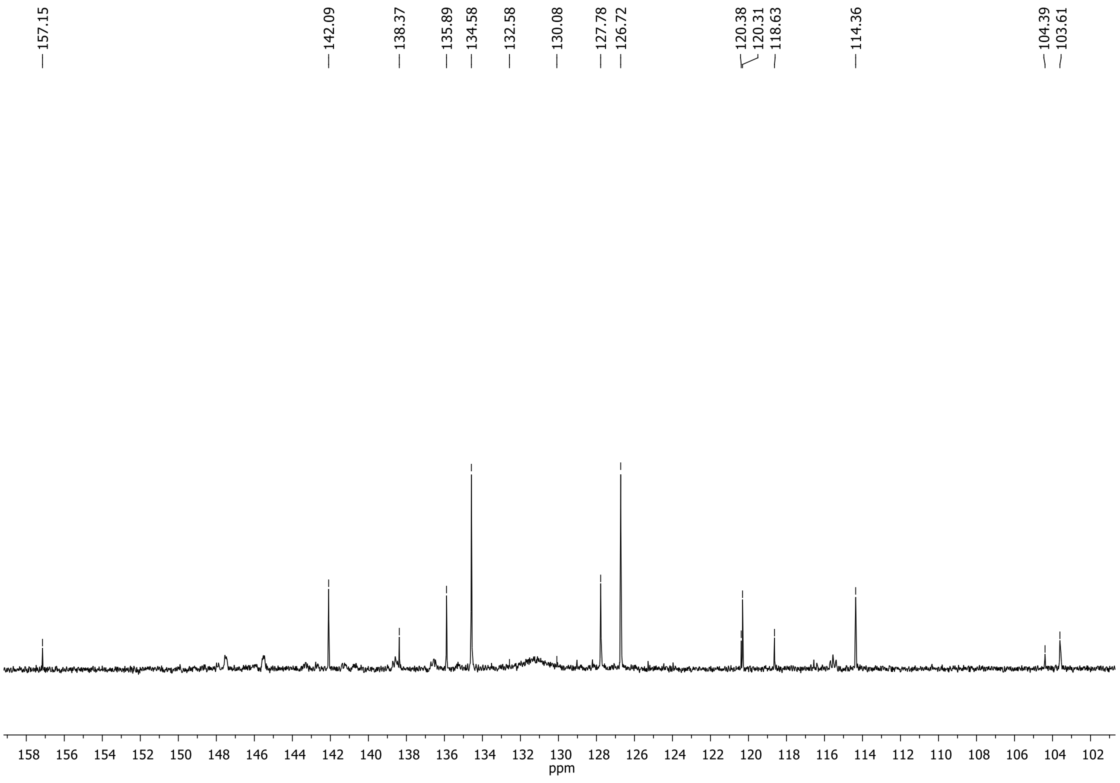

Figure S2: ^{13}C NMR spectrum of diporphyrin **3** (in $CDCl_3$).

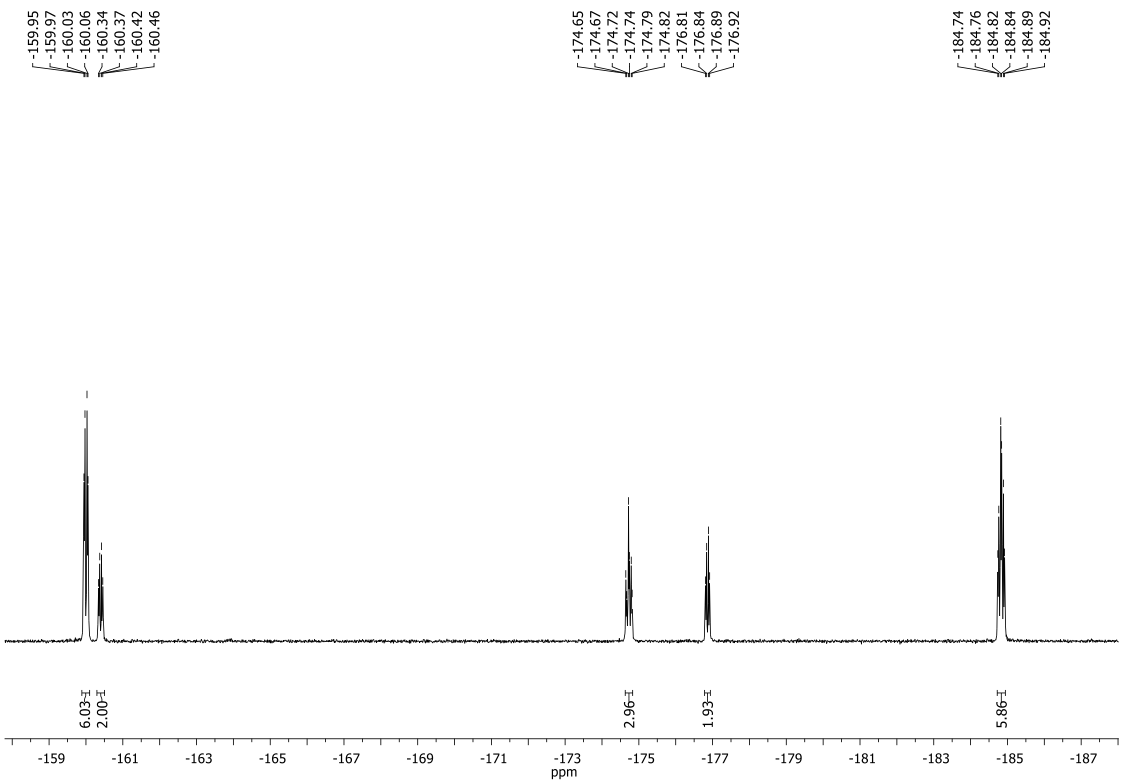

Figure S3: ^{19}F NMR spectrum of diporphyrin **3** (in $CDCl_3$).

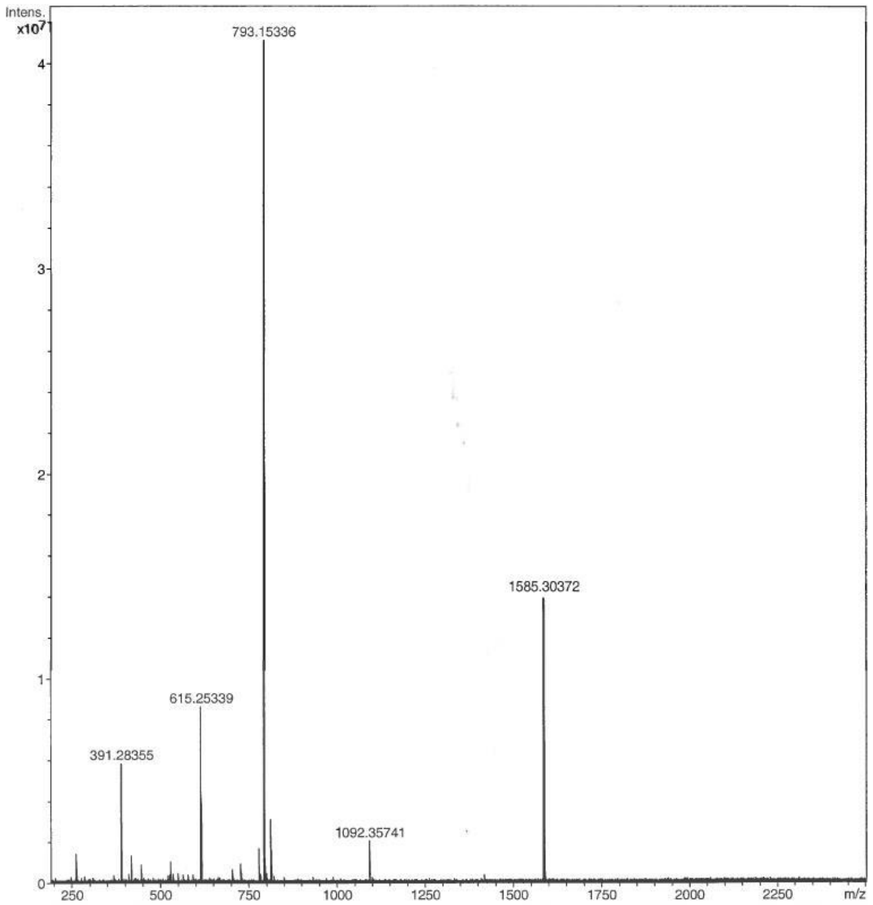

Figure S4: High-resolution electrospray ionization mass spectrum (ESI MS) of diporphyrin **3**.

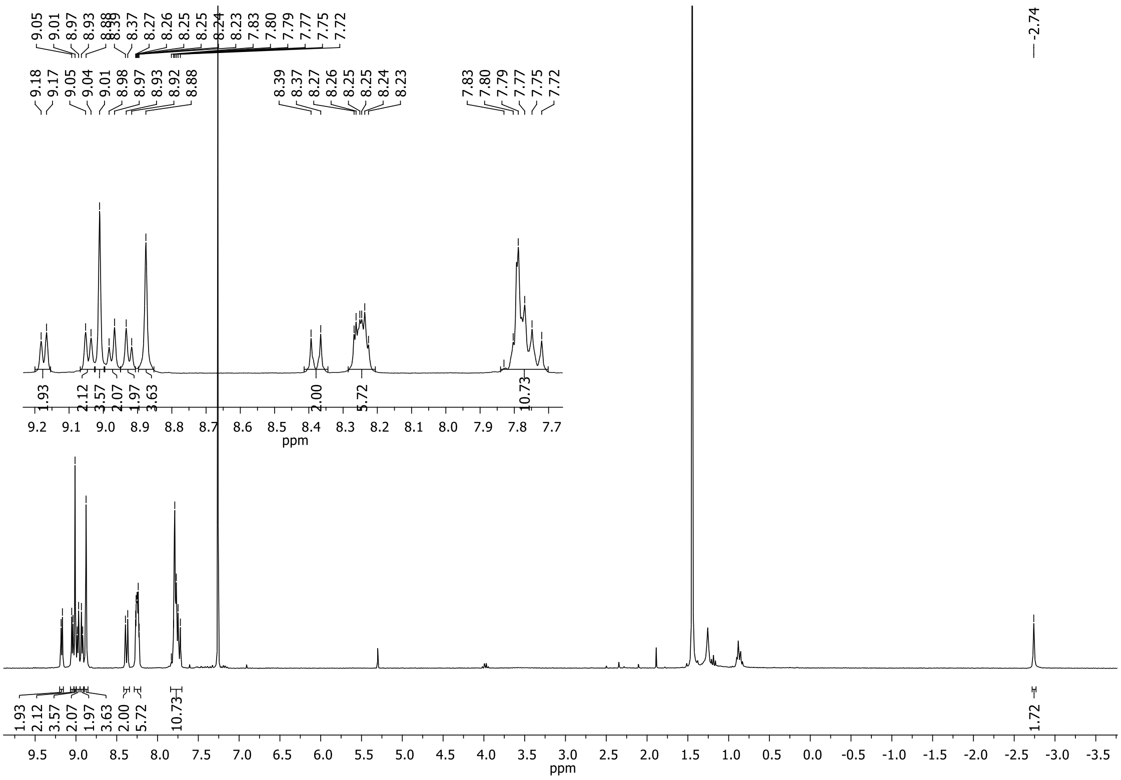

Figure S5: ^{1}H NMR spectrum of diporphyrin **4** (in $CDCl_3$).

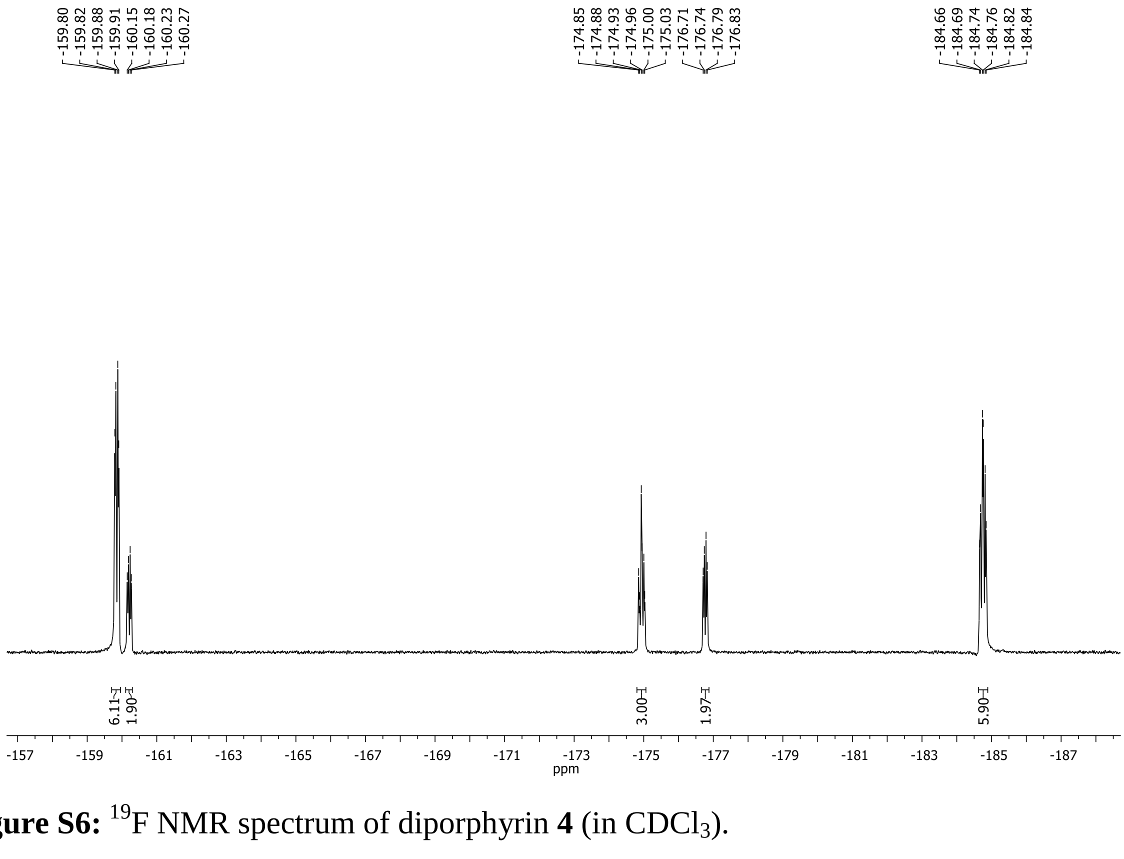

Figure S6: ^{19}F NMR spectrum of diporphyrin **4** (in $CDCl_3$).

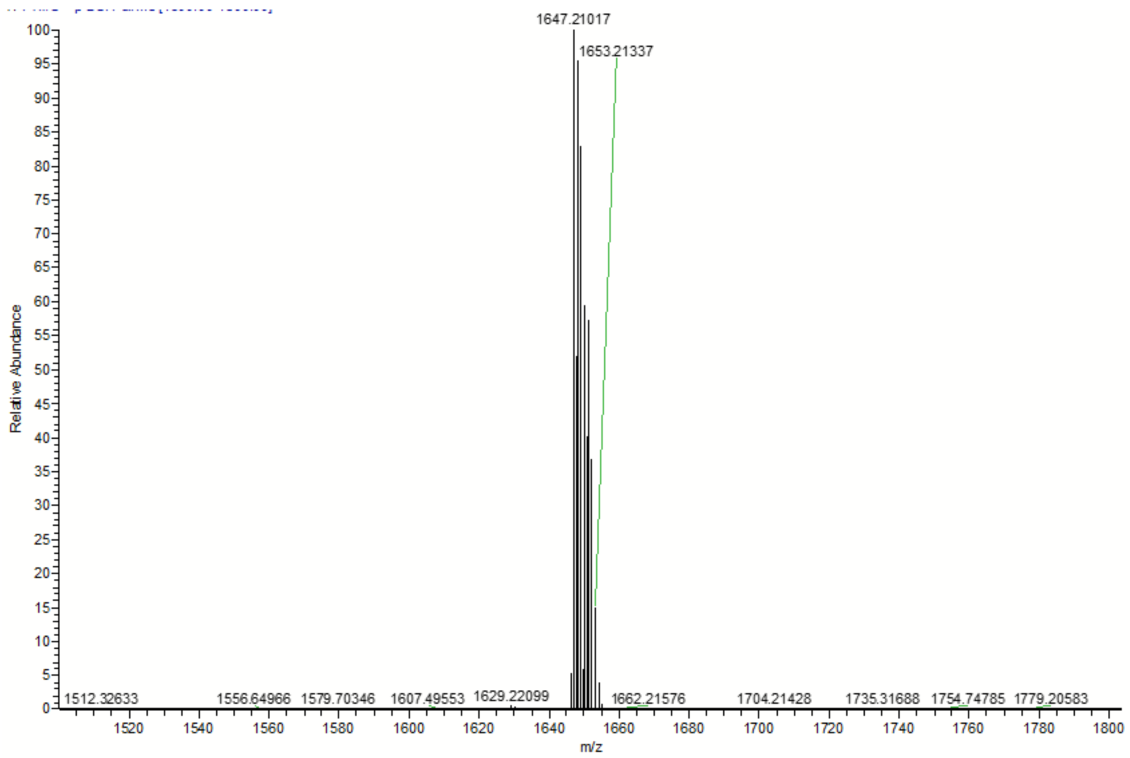

Figure S7: High-resolution electrospray ionization mass spectrum (ESI MS) of diporphyrin **4**.

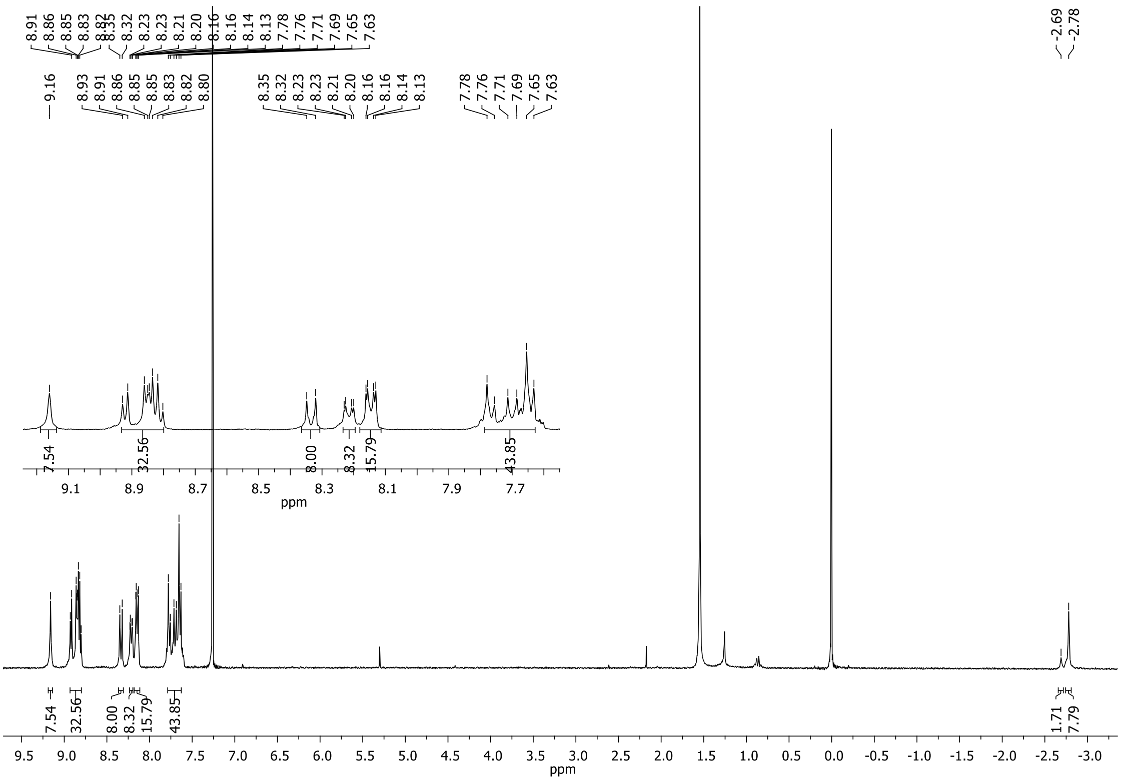

Figure S8: ^{1}H NMR spectrum of pentaporphyrin **5** (in $CDCl_3$).

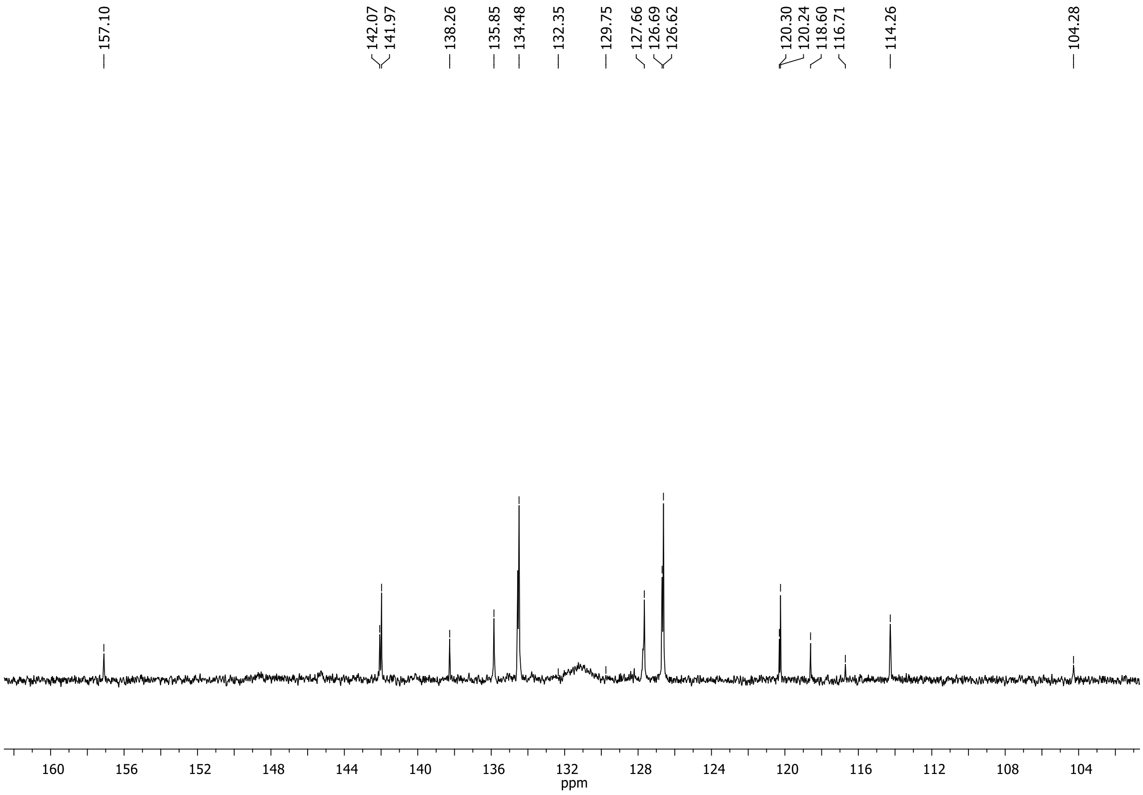

Figure S9: ^{13}C NMR spectrum of pentaporphyrin **5** (in $CDCl_3$).

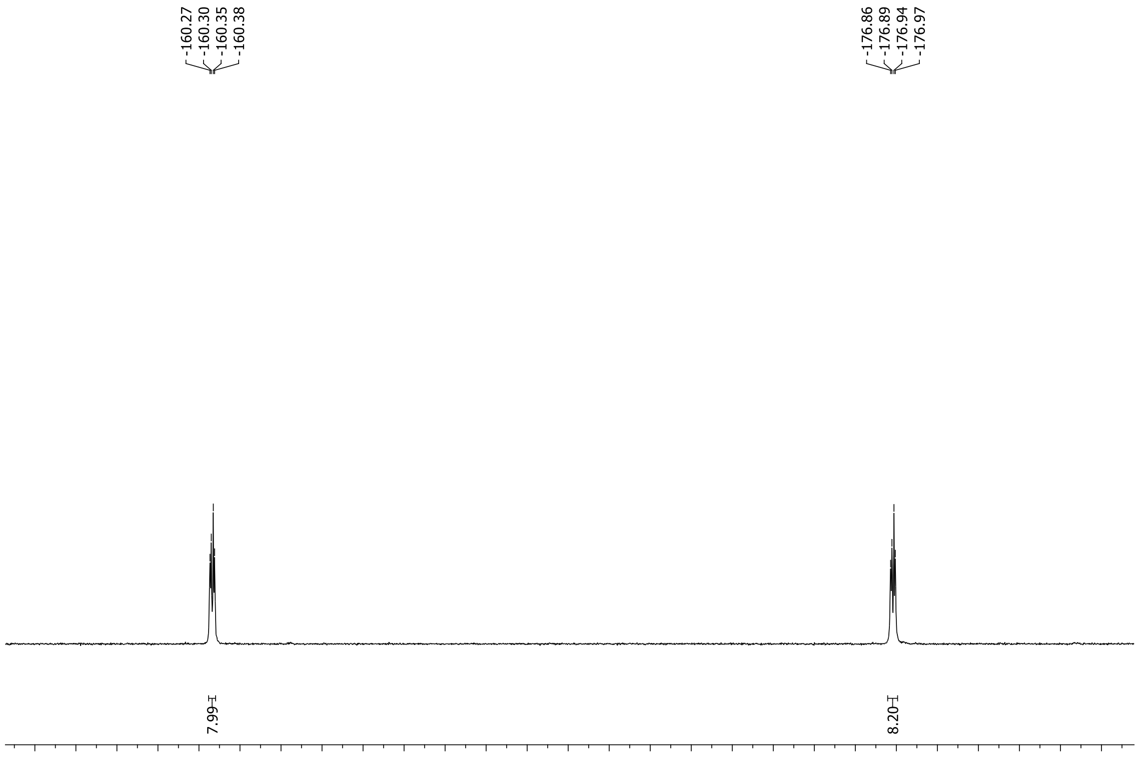

Figure S10: ^{19}F NMR spectrum of pentaporphyrin **5** (in $CDCl_3$).

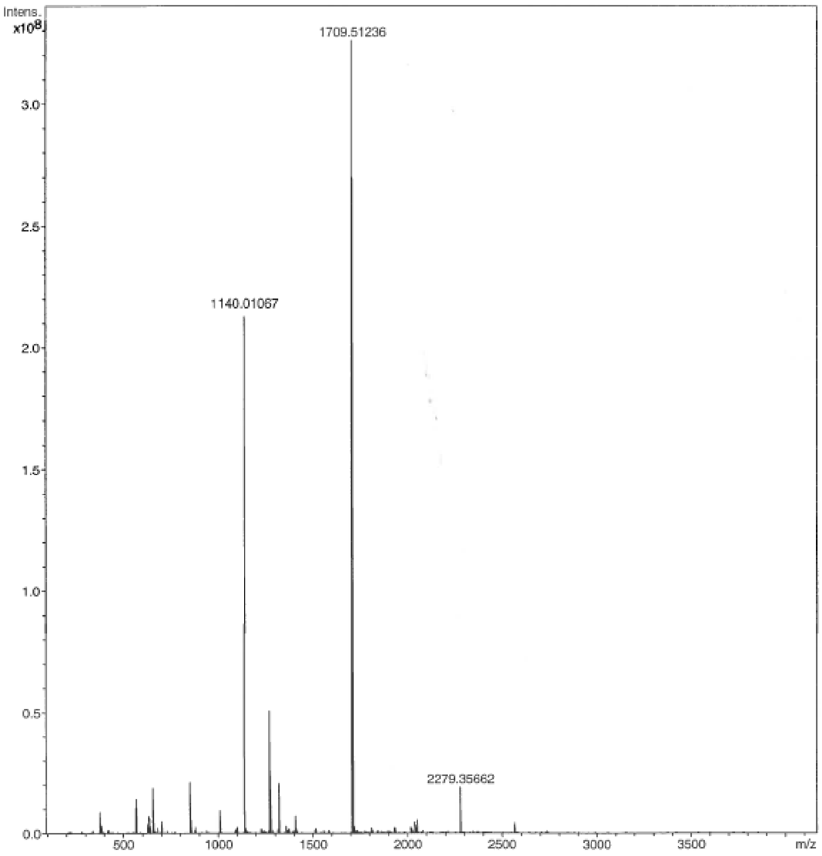

Mass Spectrum List Report

m/z	I	Res.
391.28399	3300140	155923
631.24927	4071342	96609
632.25264	1848788	98120
637.24917	6845205	92207
637.75096	7118488	93793
638.25259	3142811	95104
644.28082	6324030	94423
645.28426	3130948	94420
659.24413	18684036	91407
660.24746	8804872	92492
661.25066	2062732	92113
684.20804	1877452	89365
684.40888	2492204	84788
684.60965	1852780	86071
703.27057	4838244	86001
704.27397	2321253	88446
854.75792	6908612	68801
855.00900	16069956	70174
855.25975	21022278	70543
855.51053	17683526	69249
855.76131	11001160	67359
856.01219	5658185	67219
856.26352	2696586	66207
880.93597	2583498	70268
881.27024	2545292	69610
1009.35679	6331159	59105
1009.85827	9571105	59189
1010.35966	7625515	58152
1010.86162	3943989	58764
1100.84808	2752251	54196
1101.35018	2187466	54854
1139.00630	2784762	50890
→ 1139.34266	65527620	53858
1139.67651	168240976	54483
1140.01067	214968160	54734
1140.34528	184113008	54142
1140.68016	112920440	54104
1141.01498	55474048	52517
1141.34969	23587724	51419
1141.68418	8982168	50817
1142.01845	3276964	49847

m/z	I	Res.
1145.34431	2423258	53646
1145.67963	2628838	50244
1146.01461	2322098	50847
1146.34939	1700446	52757
1228.73081	1816881	47579
1273.49363	50677220	47202
1274.49706	50610692	47321
1274.52690	3772422	86489
1275.50022	24052774	45383
1275.53012	1858982	85448
1276.50297	7509573	45626
1277.50568	1844069	44894
1320.40020	11128110	45105
1320.90162	20923708	45389
1321.40298	19324744	45336
1321.90497	12433236	45368
1322.40665	6380897	44382
1322.90864	2500589	43810
1360.49322	2052881	43957
1360.82741	2856151	43718
1361.16160	2788317	45018
1361.49630	2144803	44285
1409.40614	4268167	37217
1409.90703	7303310	39783
1410.40821	7120021	41095
1410.90942	4646812	40648
1411.41219	2071330	40803
1515.21169	2140082	40832
1515.46230	2137655	37225
1515.71291	1863612	39036
1708.01076	3066869	33885
→ 1708.51286	84407312	36109
1708.77396	5160100	29274
1709.01196	244091952	36914
1709.26710	22365762	32562
1709.51236	326413408	36636
1709.76763	28771936	32682
1709.96169	26678380	65999
1710.01420	272657536	36864
1710.26896	18378880	31876
1710.51676	162892944	36620
1710.77176	6931616	30058

Figure S11: High-resolution electrospray ionization mass spectrum (ESI MS) of pentaporphyrin **5**.

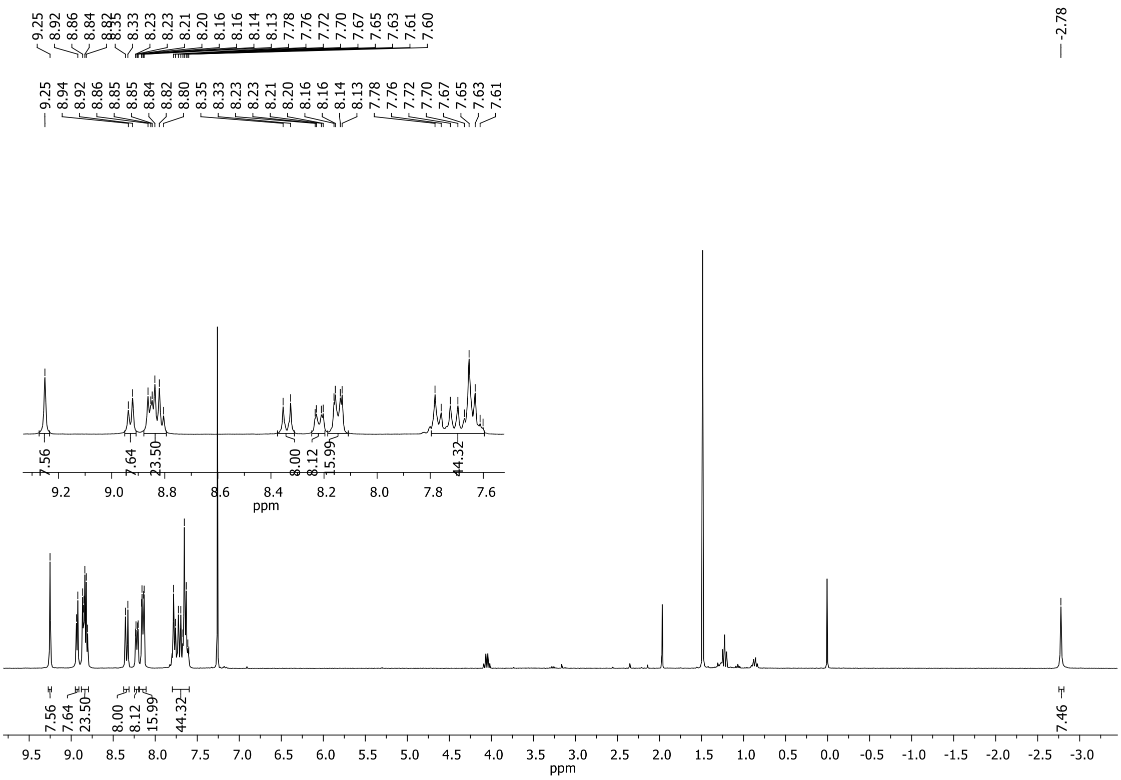

Figure S12: ^{1}H NMR spectrum of pentaporphyrin **6** (in $CDCl_3$).

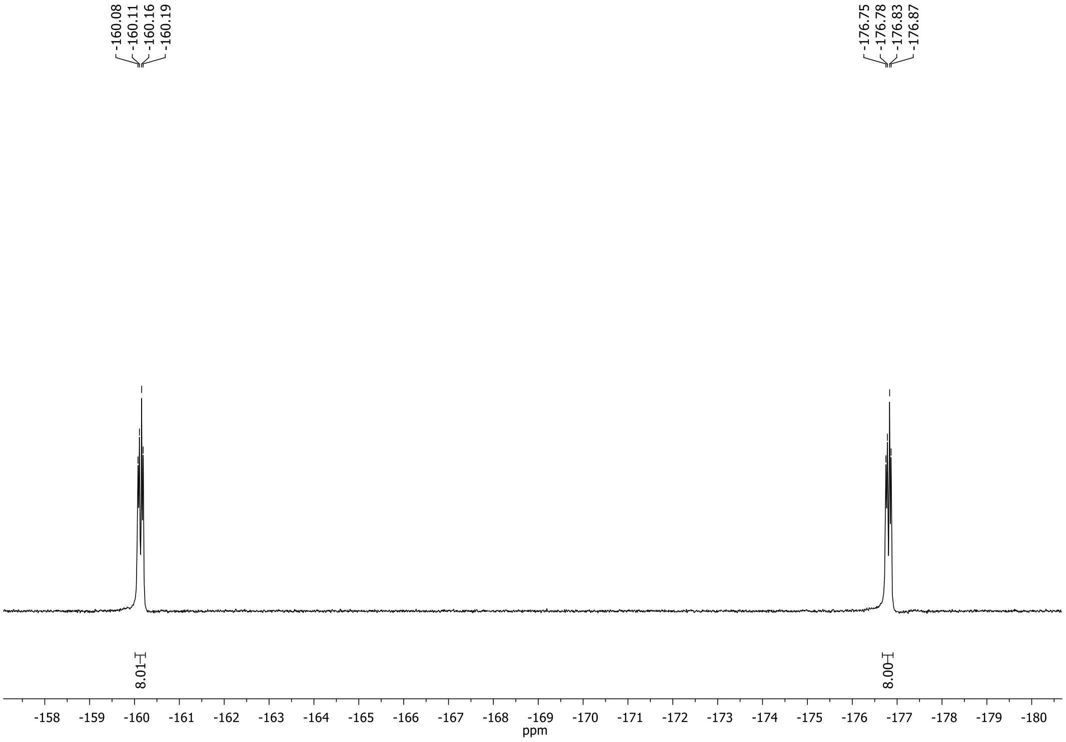

Figure S13: ^{19}F NMR spectrum of pentaporphyrin **6** (in $CDCl_3$).

2. Absorption and fluorescence titrations with PyC_{60}

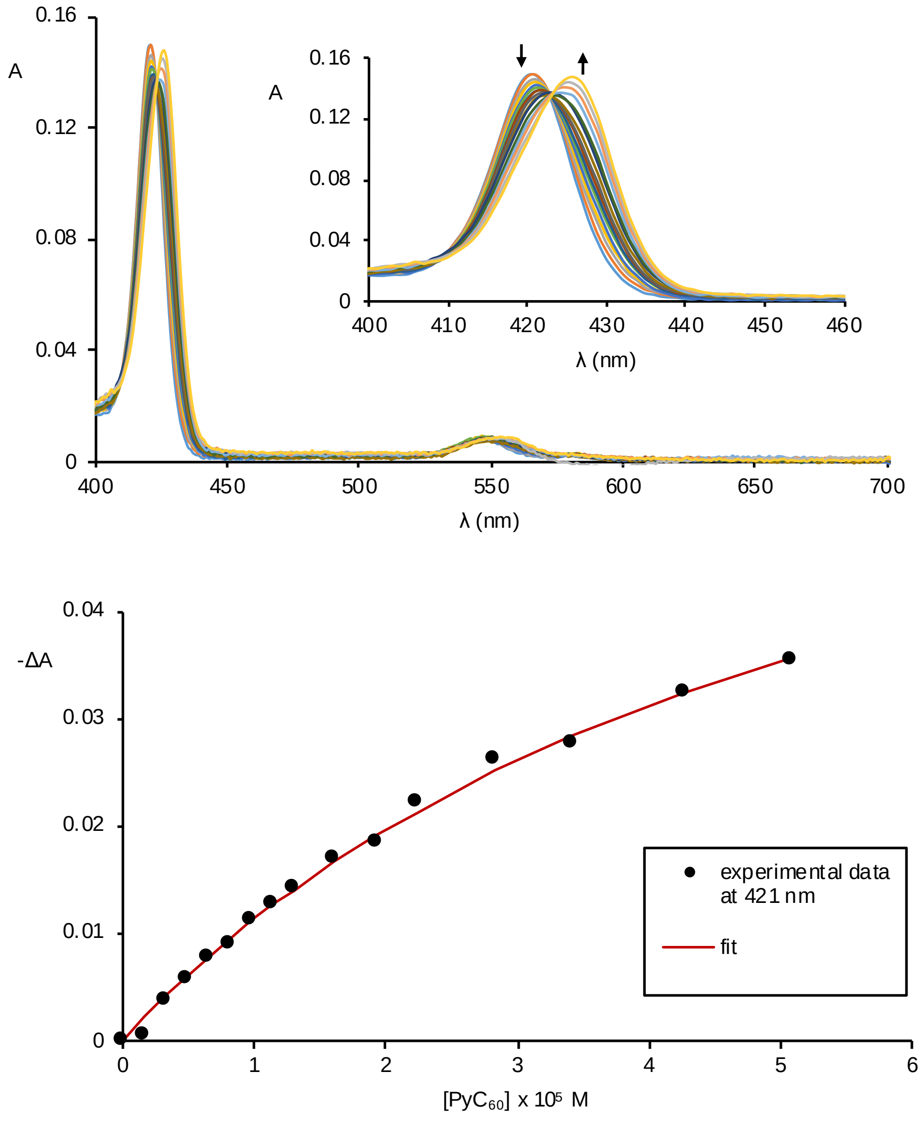

Figure S14: Absorption spectra of Zn**2** (5.0×10^{-7} M) upon addition of PyC_{60} (0–113 equiv.) in toluene at ambient temperature (upper part) and experimental data at 421 nm fitted to a non-linear 1:1 binding model (lower part).

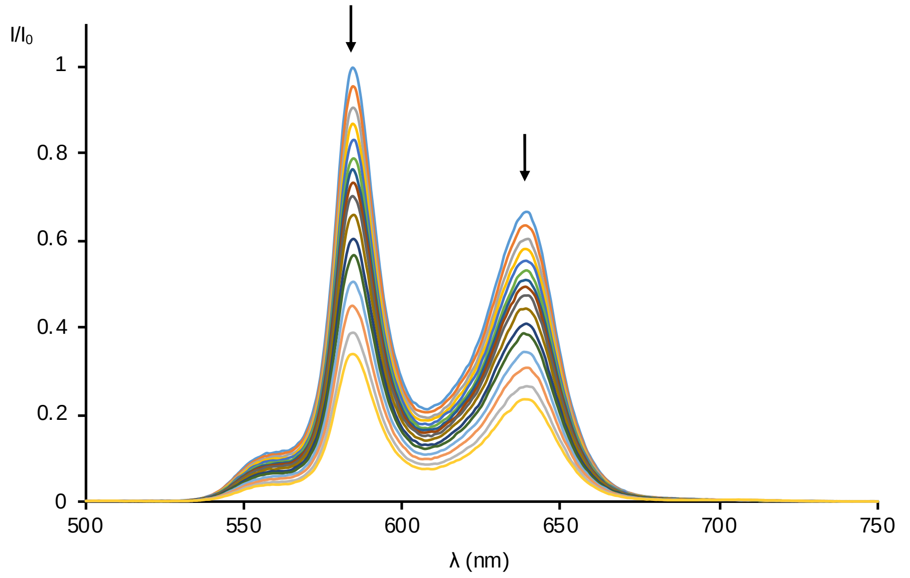

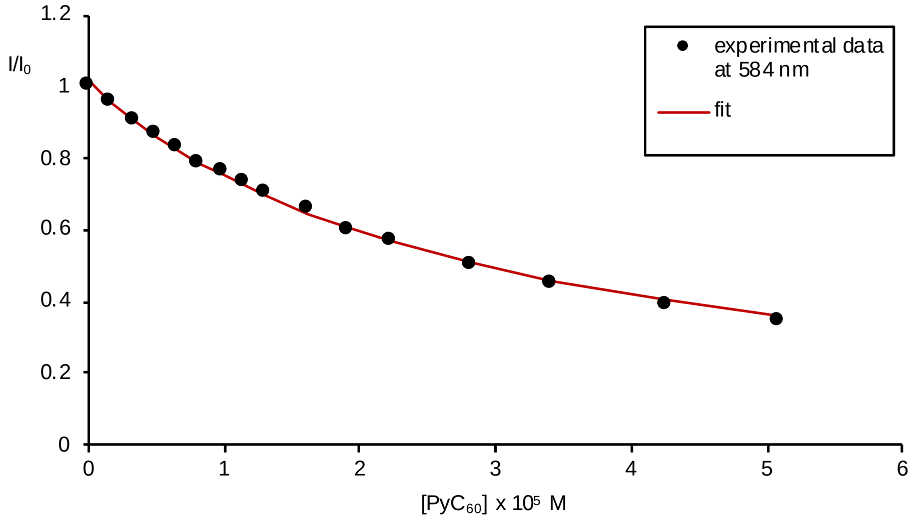

Figure S15: Fluorescence spectra (λ_{exc} = 423 nm) of Zn**2** (5.0×10^{-7} M) upon the addition of PyC_{60} (0–113 equiv.) in toluene at ambient temperature (upper part) and experimental data at 589 nm fitted to a non-linear 1:1 binding model (lower part).

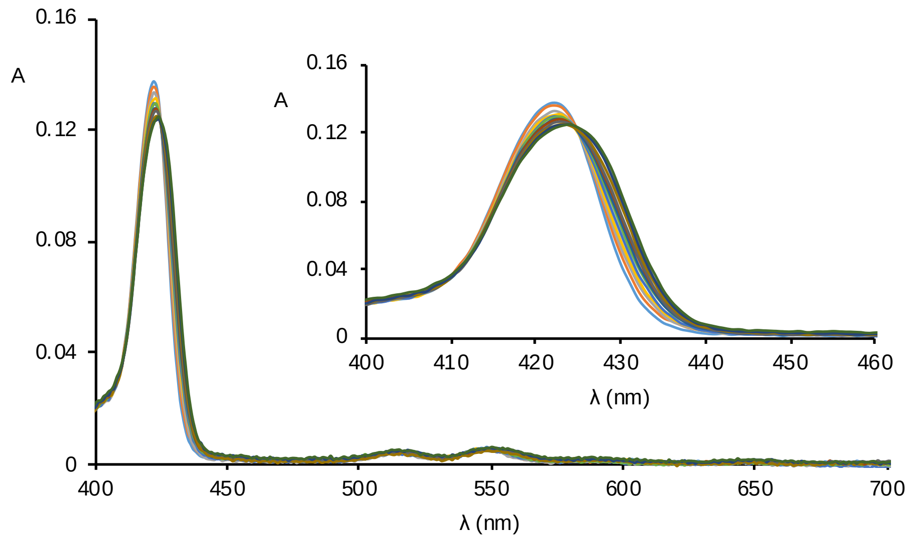

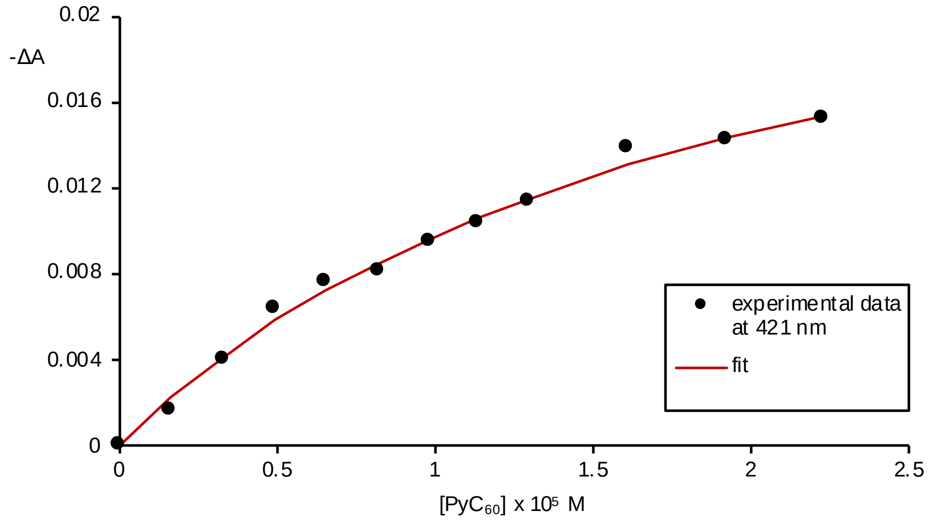

Figure S16: Absorption spectra of diporphyrin **4** (2.0×10^{-7} M) upon addition of PyC_{60} (0–116 equiv.) in toluene at ambient temperature (upper part) and experimental data at 421 nm fitted to a non-linear 1:1 binding model (lower part).

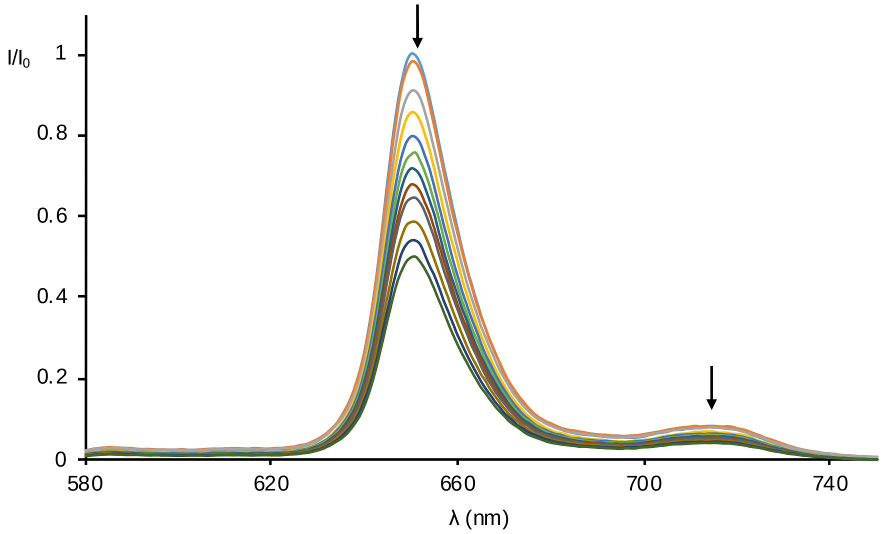

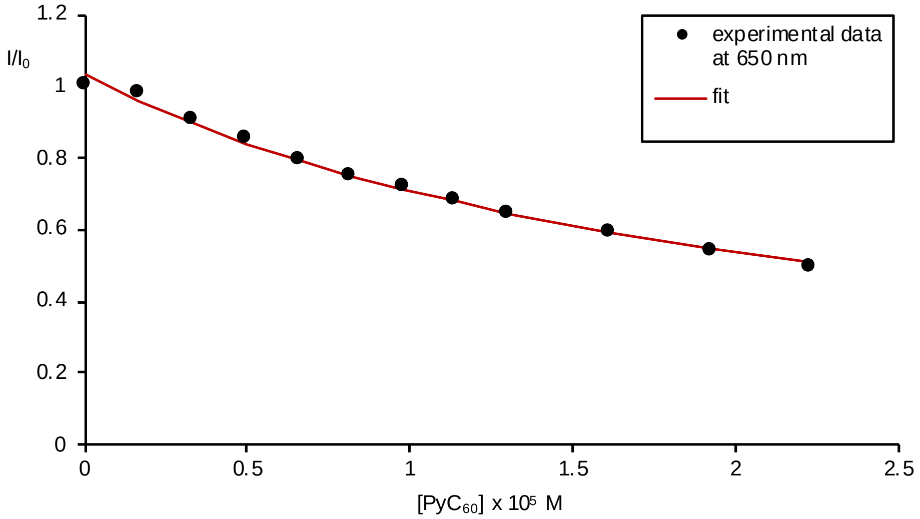

Figure S17: Fluorescence spectra (λ_{exc} = 425 nm) of **4** (2.0×10^{-7} M) upon addition of PyC_{60} (0–116 equiv.) in toluene at ambient temperature (upper part) and experimental data at 650 nm fitted to a non-linear 1:1 binding model (lower part).
